# Assessing the feasibility and appropriateness of introducing a national health insurance scheme in Malawi

**DOI:** 10.1186/s41256-019-0103-5

**Published:** 2019-05-20

**Authors:** Adrian Gheorghe, Kai Straehler-Pohl, Dominic Nkhoma, Wathando Mughandira, Denis Garand, Deliwe Malema, Alexandra Murray-Zmijewski, Andrew Kardan, Tomas Lievens

**Affiliations:** 1 Oxford Policy Management Ltd, Level 3 Clarendon House, 52 Cornmarket St, Oxford, OX1 3HJ UK; 2 Malawi German Health Programme (GIZ), Lilongwe, Malawi; 3 Health Economics and Policy Unit, College of Medicine, University of Malawi, Lilongwe, Malawi; 4 Ministry of Health, Government of Malawi, Lilongwe, Malawi; 5 Independent consultant, Regina, Canada; 6 Independent consultant, Lilongwe, Malawi

**Keywords:** Social health insurance, Malawi, Strategic purchasing, Equity, Assessment, Feasibility

## Abstract

**Background:**

In May 2015 the Malawian Ministry of Health (MOH) contacted the German Development Cooperation to seek technical assistance from the P4H Network for Social Health Protection for an “Assessment of the appropriateness and feasibility of National Health Insurance in Malawi” against two alternative options: continuing with a tax (and donor)-funded National Health Service, and introducing a purchaser-provider split without a revenue collection function.

**Methods:**

A health financing benchmarking matrix was agreed with MOH, with six domains corresponding to six objectives: revenue mobilisation, technical efficiency, equity, financial risk protection, policy coordination, and health outcomes. The assessment comprised key informant interviews with Malawian stakeholders, a review of the relevant literature and datasets, rapid assessments of the Malawi Revenue Authority (MRA) and the Unified Beneficiary Registry (UBR), and projections of the National Health Insurance Scheme’s (NHIS) revenue collection costs and benefits.

**Results:**

A key finding was that introducing NHIS in Malawi would increase revenues for health, but these would come predominantly from the formal sector and would be unlikely to cover the health sector funding gap. The performance of existing poverty identification and targeting mechanisms was not commensurate with the requirements of a NHIS. Incentives to enrol in NHI are insufficient to reach scale unless service fees be introduced, which would negatively affect equity and financial risk protection. The assessment identified the Purchaser Scenario as the most favourable reform model.

**Conclusions:**

As ever more countries look towards implementing National Health Insurance, the proposed assessment framework can provide an orientation for evidence-based policy making in the area of health financing.

**Electronic supplementary material:**

The online version of this article (10.1186/s41256-019-0103-5) contains supplementary material, which is available to authorized users.

## Background

Many countries in Africa are implementing Social Health Insurance (SHI). According to the Global Health Expenditure Database, in 2016, 22 of the 47 countries in the sub-Saharan Africa Region have had income from compulsory health insurance greater than zero (19 of the 22 are classified as SHI) [1]. At least two other countries, Madagascar and Zambia, are currently developing SHI legislation (information obtained through the P4H Network).

In Malawi, interest in SHI dates back to at least 2011 and developed out of the desire to increase domestic resource generation and reduce dependency on donor funding. Since then several working papers have been produced on (S)HI in Malawi [2–4]. The Democratic Progressive Party (DPP), who went on to win the 2014 general elections, had included in its manifesto the provision of health insurance for all public servants and a later roll-out to all in salaried employment and the informal sector [5]. In 2015, this election promise was one of four key reforms agreed between the new President, Arthur P. Mutharika, and the then Minister of Health, Jean A. N. Kalilani [6].

Following these developments, in May 2015 the Malawian Ministry of Health (MOH) contacted the German Development Cooperation to seek technical assistance from the GIZ and the P4H Network for Social Health Protection for an “Assessment of the appropriateness and feasibility of National Health Insurance in Malawi” against two alternative options: continuing with a tax (and donor)-funded National Health Service, and introducing a purchaser-provider split without a revenue collection function. Important design features of the National Health Insurance (NHI) model to be assessed were decided on by the MOH and included: (1) universal membership, with contributions from non-poor households in the civil service, formal sector and informal sector, and full subsidies for poor households; (2) a benefit package equivalent to the MOH’s Essential Health Package.

We report on the application of a methodological approach developed by Oxford Policy Management (OPM) in collaboration with MOH and GIZ for a comprehensive, multidimensional assessment of the NHI reform scenarios, combining qualitative and quantitative information. We believe the approach can be informative for practitioners and decision makers considering similar policy developments in their respective settings.

## Methods

The work was conducted in three phases over the course of the calendar year 2016. In Phase 1, four scenarios selected by the MOH and partners were assessed against the option of maintaining the status quo. In Phase 2, the MOH organised a stakeholder consultation where the assessment results were reviewed and three scenarios were retained in the analysis. In Phase 3, the three retained scenarios underwent in-depth exploration in terms of organisational design and business processes.

### Description of assessed scenarios

The NHI reform scenarios were:

- Maintain the existing institutional arrangements with purchasing through government (MOH and local government); ongoing reforms within this framework would be implemented (e.g. decentralization of health services at district level and reforming central hospitals) [6]. We refer to this scenario hereafter as the “MOH Scenario”.
- Establish a premium based NHI: collecting mandatory direct contributions from the formal sector and the informal non-poor, while fully subsidizing the poor; pooling and purchasing at national level. In Phase 1, two benefit packages were considered: one covering tertiary health care services only; and the second covering all health care services included in the Essential Health Package (EHP). In Phase 2, the tertiary care model was rejected and the EHP model retained. We refer to this scenario hereafter as the “NHI Scenario”.
- Establish a purchasing agency and separating service purchasing from service provision, either centrally or decentralized. We refer to this scenario as the “Purchaser Scenario”.

### Approach to assessment

The approach to the assessment was to investigate the extent to which each scenario would contribute to the objectives of the Malawian health system. A health financing benchmarking matrix was agreed with MOH during the inception phase, with six domains corresponding to six health system objectives: technical efficiency, equity, financial risk protection, policy coordination, health outcomes, and revenue mobilisation. Populating the matrix with evidence for each scenario-objective pair results in a dynamic qualitative analysis showing how each NHI scenario is expected to impact on each objective. A series of narrative summaries discussing the likely impacts of each scenario were constructed, one for each of the six objectives and an overarching synthesis. All summaries and their supporting evidence were discussed, refined and validated during the stakeholder consultation in Phase 2.

The assessment comprised the following components: key informant interviews with Malawian stakeholders; a review of the relevant national and regional published literature, reports and datasets; a rapid assessment of the systems relevant for collecting contributions and targeting subsidies, i.e. Malawi Revenue Authority (MRA) and the Unified Beneficiary Registry (UBR) of social protection schemes; and conducting projections of revenue collection costs and benefits. We outline methods for each below, with further details in the Additional file 1.

### Assessment components

Key informant interviews were conducted with representatives of ministries, government organizations, civil society and private sector organizations (Additional file 1: Appendix 1). The key informants were identified in consultation with the MOH on the basis of their technical knowledge, policy experience and institutional affiliation, with a view to obtaining a representative and ample range of expert accounts. The interview guide focused on the following topics: the priority level of health insurance reform; mapping the ongoing policy initiatives and how they could relate to the introduction of health insurance reform; perceived priorities for additional funding in the health sector; to sound out their views on which institutions and individuals could be championing this reform. One researcher took detailed interview notes, which were transcribed and analysed using thematic analysis.

The literature review comprised a PubMed search of the peer-reviewed literature (indicative search terms “health financing”, “health insurance”, “strategic purchasing”) published since 2005 with a focus on sub-Saharan African countries and an exploration of the institutional grey literature (e.g. WHO, World Bank, UNICEF). Additional sources specific to Malawi were identified with the support of MOH and the P4H network.

The rapid assessment of the MRA drew on interviews conducted with MRA staff, primarily directors of, or senior staff from, the following divisions: Policy, Planning and Research; Modernisation; Domestic Tax; Human Resources; and Information and Communication Technology. Main interview topics concerned the present capacity of the MRA to administer NHIS and collect premiums for both the formal and informal sector; and the plans in place with potential to overcome challenges in identifying and collecting premiums from the informal sector. Interviews were complemented with financial projections informed by current and hypothetical organisation structures of the MRA (Additional file 1: Appendix 4).

The rapid assessment of the Unified Beneficiary Registry (UBR) focused on the institutional setup and evidence on efficacy of the poverty targeting mechanisms of two key programmes with potential relevance to the NHIS, namely the Social Cash Transfer (SCT) and Public Works Programme (PWP) under Malawi Social Action Fund (MASAF 4). The UBR aims at providing a single source of information on households eligible for the SCT, the PWP and other social support services. The assessment included: a desk review of programme documents on the SCT, PWP, MVAC and FISP and literature on targeting under other programmes in Malawi and globally; key informant interviews with national-level stakeholders in the area of social protection; group discussions with programme officials in two districts; and an analysis of the Integrated Household Survey 3 (IHS3) dataset to review the performance of the Proxy Means Test (PMT) used in Malawi to target the poorest sections of the population.

The NHI reform scenarios were integrated into a health sector expenditure and revenue model, which estimated the overall effect of the reforms in terms of financing the health sector as a whole (details in Additional file 1: Appendices 2–4). At the centre of the model are yearly population projections by age bands, which inform estimates of payments to providers based on the expected evolution of the disease profile and revenue estimates. We projected population using Demographic and Health Survey (DHS) 2015–2016 data. A health expenditure index was developed for four age bands based on the consultant’s previous work on the benefit package in Kazakhstan and on Malawi’s population age structure, taking into account that newborns and the elderly consume relatively more resources: 0–1 years – 3.0; 1 to 19 years – 0.6; 20 to 59 years – 0.9; 60+ years – 2.0. Health expenditure projections were primarily informed by the health expenditure index linked with population structure, also taking into account inflation and service improvements. Inflation data were informed by the World Economic Outlook (WEO) Database and averages of subsequent years were taken to reflect the structure of Malawi’s fiscal year, which runs 01 July – 30 June (e.g. inflation in fiscal year 2015/2016 was assumed to be the average of 2015 and 2016 inflation in the WEO Database).

Health sector revenues were informed by the National Health Accounts 2012/2013–2014/2015 data and included donor funding, incorporated inflation and yearly % increases in allocations to health proportional with population growth (2.1 to 2.3% per annum). Three NHI enrolment scenarios were modelled: a realistic enrolment scenario assuming 5% of the informal non-poor (1% of the total population) would enrol in the NHIS by 2021/22 (base-case scenario); an optimistic enrolment scenario assuming 25% of the informal non-poor (5% of the total population) would enrol; and an exceptional enrolment scenario, assuming 40% of the informal non-poor (8% of the total population) would enrol. For the formal sector, it was assumed that 100% would be enrolled from the start; the same was assumed for the informal poor, with the Government of Malawi paying their contributions in full (i.e. a 100% subsidy). An NHI contribution of MWK 3000 per individual was assumed, based on international experience of membership premiums of ~ 1% of average per capita incomes. A recent estimate of willingness-to-pay for insurance in Malawi was even lower at just above MWK 3000 for coverage of an extended family [7].

We also estimated the cost of a NHI scheme in Malawi, including the set up and running costs of a national agency administering the health insurance funds, the cost of regulating NHI, the cost of purchasing and the cost of revenue collection through the MRA. Details of the assumptions and calculations, e.g. assumed staff structure for NHI and purchasing agency, are in Additional file 1: Appendices 3-4.

## Results

First, we present key findings of the assessment under each health financing objective, then the results of the cost and revenue projections and then the populated benchmarking matrix.

### Technical efficiency

The key areas of inefficiency in the Malawian health sector were identified in previous health sector strategic documents. They include: medicines (e.g. under usage of generics drugs; over prescription of antibiotics; leakages in pharmaceutical supply and distribution); service delivery (e.g. underutilization of existing inpatient bed capacity); and human resources for health (e.g. inequitable distribution of health care staff in relation to health need; inadequate training and retention mechanisms). The MOH has been implementing reforms to address some of these inefficiencies, such as: health services decentralization; increasing the managerial autonomy of service providers; contracting health services from non-governmental organisations; granting of autonomy to the Central Medical Stores Trust (CMST); and reviewing the essential health package on principles of cost-effectiveness. The implementation details of these reforms were essential for all scenarios in this analysis, but their cumulative effect was difficult to quantify.

Based on other countries’ experience of transition towards strategic purchasing (e.g. Turkey, Thailand), there is a justified expectation that separating purchasing from service provision increases health spending efficiency under the NHI and the Purchaser Scenarios. This is due to potential positive effects stemming from: the contractual relationship between purchasers and providers that can balance financial risks between the two; improving health treatment protocols; and improving financial management. There was, however, limited evidence assessing directly the impact of introducing NHI on technical efficiency aspects of the health system to differentiate between the Purchasing Scenario and the NHI Scenario.

### Equity

Interviewees framed equity in access to health services in Malawi along several dimensions:

- Malawians vs Non-Malawians: in border districts, Non-Malawians access services financed by taxpayers in Malawi. Interviewees estimated that up to 20% of services are delivered to non-Malawians.
- Informal sector vs formal sector: given the current health financing arrangements, health services are financed almost fully by those in the formal sector through personal income tax and VAT on goods and services traded in the formal economy. The non-poor in the informal sector, estimated at 20% of the population, do not contribute to their full potential.
- Poor vs non-poor: While access to health care is theoretically free, stakeholders widely agreed that this is not true in practice. Direct medical (e.g. fees), direct non-medical (e.g. transport costs) or informal payments are common, which results in poor people benefiting less than the well off from tax-funded service provision.

The issues of the informal/formal sector and the poor/non-poor can only be evaluated together, since only the non-poor part of the informal sector is expected to contribute to financing health care in Malawi. Making non-poor households in the informal sector contribute (but not the poor) could be done, in principle, by effectively identifying either of the groups: identify the non-poor income-earning households through the MRA and charge a contribution (be it an NHI contribution or tax); or collect the contribution of the non-poor informal sector through a “service access fee” from which poor households would be exempted based on identification information in the UBR. As such, without a “service access fee” and in the absence of other public enforcement mechanisms of a legal mandate for enrolment, there would be little incentive for a non-poor household to pay for using services it could also access for free – however, introducing fees would likely have a strong negative impact on equity, discussed below.

The rapid assessment of the targeting schemes under the UBR identified a number of challenges in the design and implementation of the targeting models. The predictive power of the targeting models underlying the UBR was rather weak: 60% of households in the poorest quintile were not correctly identified as such, while 44% of those in the richest quintile were wrongly identified as not belonging to this group. Such targeting errors would likely also occur around the suggested cut-off point of 50% of the population in an NHI model. This means that many households eligible for a full Government subsidy towards their NHIS contribution would not receive it and, thus, face an additional financial barrier to accessing services. As such, it seemed inappropriate to use these models for determining fee paying ability of households and, as a result, determining their rights to accessing insured health services.

None of the reform scenarios had an inherent advantage in limiting access to services for Malawian nationals. Consequently, introducing NHI combined with service access fees could not be recommended from an equity perspective. Introducing a purchasing agency may affect equity positively as a purchaser-provider split would be expected to increase service quality, including in health centres and district hospitals, to which the poor have better access than to tertiary centres. This improvement would depend, though, on integration with other reforms, e.g. increase hospitals’ managerial autonomy.

### Financial risk protection

Introducing the Purchaser Scenario would not directly affect financial risk protection; indirectly, if coupled with other policies (e.g. greater provider autonomy, introducing output-based provider payment mechanisms), in the long term it may reduce providers’ dependence on user fees and informal payments, with potential positive impacts on patients’ financial risk protection. The NHI Scenario, on the other hand, can protect to an extent against catastrophic and impoverishing health expenditures. Other African experiences to this end include Ghana, Nigeria, and Rwanda.

The introduction of NHI can increase financial risk protection for its members; however, the conditions in Malawi are not met to effectively increase financial risk protection across the population. First, the introduction of service access fees to stimulate NHI enrolment would detrimentally affect risk protection, especially of the poor, as outlined above. Second, NHI must be coupled with other measures. For example, it was suggested during consultations that if health workers’ salaries do not increase and provider management practices does not improve, informal payments are likely to continue and quality of care would remain the same, which would cancel any financial risk protection effect. As such, the NHI Scenario did not appear to improve financial risk protection relative to the MOH Scenario under current conditions.

### Policy coordination and resource allocation

The separation of service purchasing from service provision (NHI and Purchaser Scenarios) would lead to substantial additional complexity in the health sector governance and regulatory arrangements. The separation entails that these functions would be managed by different entities, a departure from the current arrangement where the MOH funds, purchases and provides a large share of health services. First, there would be more actors in the health system performing different functions, which raises the need for effective institutional coordination. Second, in order for a purchaser-provider split to improve efficiency, further policies often need to be enacted (e.g. revisiting provider payment mechanisms), which raises the need for effective policy coordination.

The separation of purchasing from service provision automatically leads to stewardship as a separate function in order to manage the new stakeholder relationships. From this perspective, all three NHI scenarios require efforts for coordination from all participating institutional actors – service purchaser(s), the government, and service providers – with the government, usually through the MOH taking on an active stewardship role.

Ensuring coherence in purchasing arrangements is likely to be an important challenge. At the moment in Malawi claims reimbursement from Service Level Agreements (SLAs) with CHAM facilities are delayed due to incomplete invoicing by CHAM, long and multiple checks for correctness at district and central (Ministry of Health and Ministry of Finance) levels. As such, more streamlined processes would be necessary.

### Health outcomes

Malawi’s disease profile is complex; however, the disease burden is comparable with that of neighbouring countries. Global Burden of Disease 2015 data confirm that Malawi’s disease burden is dominated by HIV/AIDS (19% of deaths) and other common infectious diseases, while non-communicable diseases are on the rise [8]. Improving health outcomes across the board would be best served by a balanced benefit package that emphasizes prevention and primary care delivery.

NHI can be a key reform in improving population health outcomes, but not in isolation. There are indications from African examples that the introduction of national health insurance schemes improves utilization of health services [9, 10], but the impact on health outcomes is not fully conclusive.

The transition towards active purchasing of services through the introduction of a purchaser-provider split common to the NHI and Purchaser Scenarios creates the premise for improving the quality of services, leading to better outcomes. However, the differential density of providers between rural and urban areas is likely to allow selective contracting based on quality only in urban areas because there are too few rural providers in any given area to choose from.

### Revenue and cost projections

The rapid assessment of the MRA showed that if the NHIS was to focus on the formal sector alone, revenue collection could be accomplished within current capacities and IT systems. The cost of establishing a Formal-Sector-only NHIS department in MRA would be approximately MWK 43 million in the first year, rising to MWK 54 million in year five of operation (or 0.2% of additional MRA budget). One area of investment needed, other than staff salaries and equipment, would be training staff within this new department on the peculiarities and importance of collecting NHI premiums. However, MRA did not and would not have in the near future capabilities to track informal sector individuals to collect NHI premium payments. Based on a simplified organizational design of the revenue collection function and on an expected coverage of 5% of the informal non-poor (1% of the population), by 2021/2022 annual premium collection would be MWK 601 million at an annual cost of MWK 150 million.

Additional costs would arise from identifying the 50% of Malawian households meant to be eligible for a full Government subsidy to NHI membership due to their poverty. Currently, only between 22.5 and 50% of households are formally assessed for their poverty status.^1^ Reliably distinguishing between those below the 50% threshold and those above would require assessing well above 50% of all households and the costs of targeting 50% of the population in all districts within the country, using the current targeting models feeding the UBR, has been estimated to be approximately MWK 12.9 billion, which is equivalent to 1.4% of total government expenditure and 0.6% of GDP.

The MRA also did not have the systems in place to cost-effectively identify informal sector businesses from which NHIS revenue collection could be collected, but plans were in place to strengthen these capacities through ICT improvements; connections to local-level registries and information; and administering other levies.

As such, the NHI Scenario has the potential to raise additional funding for the health sector. However, this funding would come largely from the formal sector already taxed. Collecting NHI premiums directly from the population appears to be expensive relative to raised revenue. This means that NHI would not be very effective if the main objective were to involve the informal sector in financing health care.

**Fig. 1:**
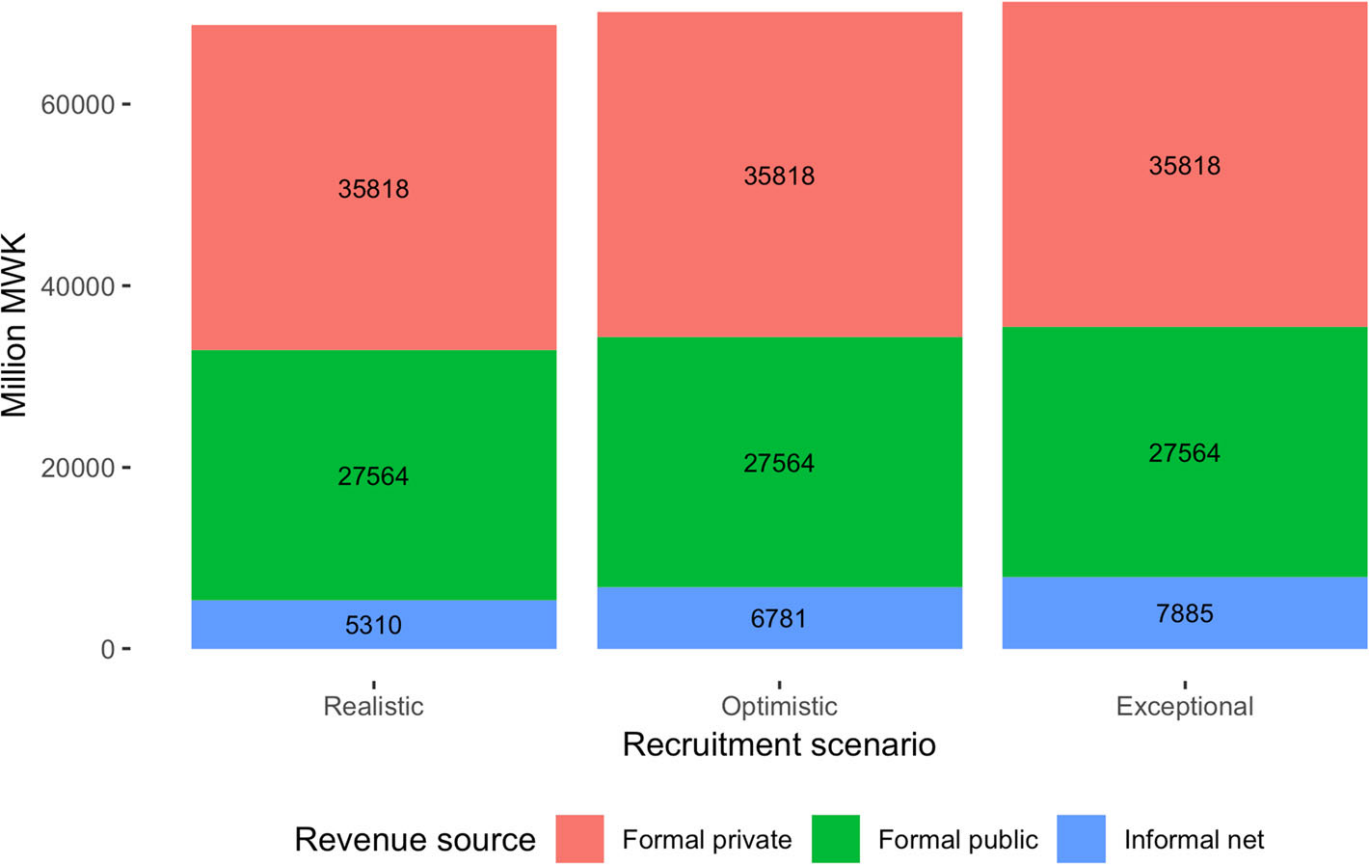
Projected total net NHIS revenue, by expectation of NHIS enrolment (2021/2022)

The model projected that total net revenue generated by the NHIS from both formal and informal sectors (including user fees) to range between MWK 68 and 72 billion by 2021/2022 (Fig. 1). Assuming 100% coverage in the formal sector, between 89 and 92% or revenue would come from the formal sector, depending on the assumed coverage rate in the non-poor informal sector. Importantly, under the assumption that the formal sector would not be burdened with additional costs, as directed by MOH, the revenue from the formal sector is not new revenue. It would simply be earmarking general government expenditure for health, e.g. by turning income taxation into an NHI contribution (assumed to be 3% each for employer and employee).

The total annual cost of administrating the NHI would amount to approximately MWK 11 billion, after an initial frontloading of the costs of an NHIS communication campaign that would bring the costs during the first year up to MWK 14 billion (Table 1). Relative to the total projected population of Malawi, this is equivalent to about MWK 550 per individual per year.

**Table 1:** Total revenue, administrative costs and net effects of NHI Scenario (MWK million)

Revenue by source / Cost by function		2017/18	2018/19	2019/20	2020/21	2021/22
Income		3718.7	4131.2	4564.2	5022.9	5514.6
NHI contributions (informal sector)^a^		552.9	614.3	678.7	746.9	820.0
User fees^a^		3165.7	3516.9	3885.5	4276.0	4694.6
Cost (by function)	Source/details	14,182.5	10,376.7	10,724.2	10,843.8	11,204.4
NHIS membership database	NHIA NHIS database	7078.5	7114.5	7269.3	7443.6	7598.4
Quality Assurance	NHIA quality assurance + Purchasing compliance	326.2	333.7	354.3	377.0	399.2
NHIS marketing	NHIS communication campaign + NHIA communication staff	4626.1	729.2	789.9	852.5	916.0
Contracting with service providers	Purchasing contracting	301.5	315.6	330.7	345.3	370.6
Insurance claims management	NHIA claims management	463.1	471.2	501.4	535.2	565.4
Other NHI and purchasing functions	NHIA other functions + Purchasing research + Purchasing other functions	662.3	664.2	687.6	687.0	701.6
Revenue collection	Formal sector revenue collection (MRA)	43.0	44.2	46.2	49.9	53.7
	Informal sector revenue collection (NHIA)^a^	138.2	153.6	169.7	186.7	205.0
Insurance regulator	Insurance regulator	12.9	13.3	13.9	15.0	16.1
Health facilities processing NHI claims		530.6	537.4	561.3	351.7	378.4
Cost (million USD)		18.9	13.8	14.3	14.5	14.9
Projected population		18,431,195	18,831,715	19,232,229	19,632,747	20,033,264
Cost per capita (USD)		1.0	0.7	0.7	0.7	0.7
Cost per capita (MWK)		769.5	551.0	557.6	552.3	559.3
Net revenue		−10,463.8	− 6245.5	− 6160.0	− 5820.9	− 5689.7

The allocation of NHIS costs to key functions is indicative only

^a^Realistic coverage scenario (5% of informal non-poor population)

Running the purchasing agency would cost about MWK 450 million annually by 2021/22 (Table 2). It was assumed that the agency operates at 100% capacity from the first year and covers 100% of eligible health service providers. About 70% of running costs would be staff salaries and benefits. These costs would also be incurred in the Purchaser Scenario.

**Table 2:** Total revenue, administrative costs and net effects of Purchaser Scenario (MWK million)

Function	2017/18	2018/19	2019/20	2020/21	2021/22
Income	0.0	0.0	0.0	0.0	0.0
Contributions	0.0	0.0	0.0	0.0	0.0
User fees	0.0	0.0	0.0	0.0	0.0
Cost (by function)	368.1	385.0	403.2	420.9	451.7
Contracting	301.5	315.6	330.7	345.3	370.6
Research	15.9	16.6	17.4	18.2	19.5
Compliance	47.6	49.8	52.2	54.5	58.5
Other functions	3.1	3.0	3.0	2.9	3.1
Net revenue	− 368.1	− 385.0	− 403.2	− 420.9	− 451.7

Table 3 combines the results of the revenue mobilization and the technical efficiency effects on the financing of the health sector, it can be seen that for the next five years, the Purchaser Scenario is expected to have the largest positive effect.

**Table 3:** Comparison of financial effects of the reform scenarios (MWK million)

Total effect of Scenario		2017/18	2018/19	2019/20	2020/21	2021/22
MOH		0.0	0.0	0.0	0.0	0.0
NHI (all effects)		1050.3	18,942.0	30,512.3	44,068.1	54,195.9
positive effects:	additional revenue	3718.7	4131.2	4564.2	5022.9	5514.6
	efficiency gains	11,514.1	25,187.5	36,672.3	49,889.0	59,885.7
negative effects:	administration costs	−14,182.5	−10,376.7	−10,724.2	−10,843.8	−11,204.4
Purchaser (all effects)		11,146.1	24,802.5	36,269.0	49,468.1	59,434.0
positive effects:	additional revenue	0.0	0.0	0.0	0.0	0.0
	efficiency gains	11,514.1	25,187.5	36,672.3	49,889.0	59,885.7
negative effects:	administration costs	−368.1	−385.0	−403.2	−420.9	−451.7

**Fig. 2:**
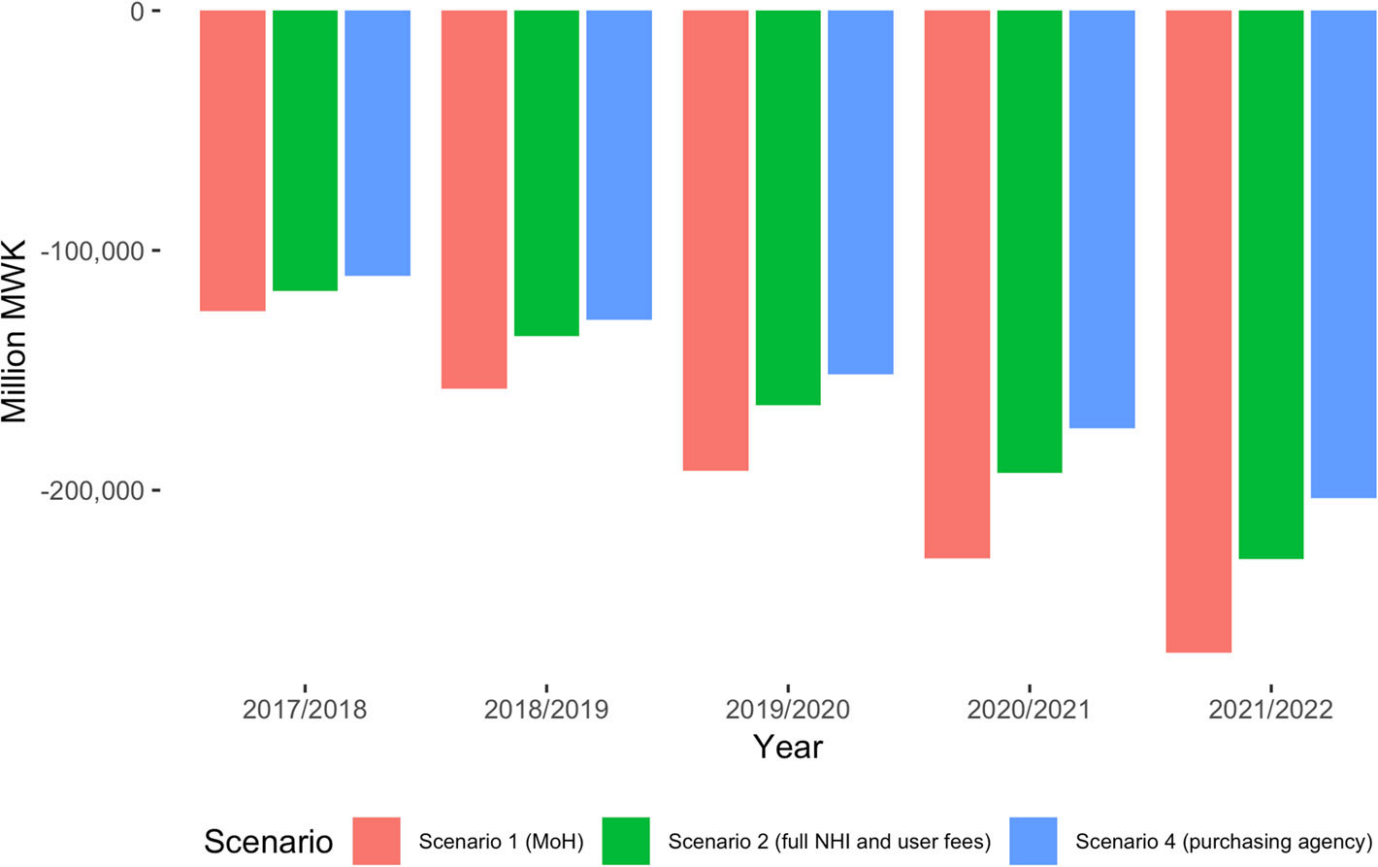
Impact of reform scenarios on the health financing gap

Under all reform scenarios, Malawi will continue facing a funding gap that will require external financing. Net revenue projections (revenues minus expenditure) suggest that the funding gap would slowly increase for all scenarios over the next few years (Fig. 2). Under the status quo (MOH Scenario), the gap would be of about MWK 250 billion by 2021/2022. The NHI and Purchaser Scenarios each close the gap partially to below MWK 230 billion – mostly driven by the projected efficiency gains.

All scenario estimates include additional MWK 4 billion annual revenue from a combination of three levies that could, based on work on a “Health Fund” by the World Bank from October 2016, realistically be earmarked for the health sector.^2^ Even when accounting for the potential efficiency gains associated with purchasing, worth approximatively MWK 54 billion under scenario 2 (full NHI) by 2021/2022, a funding gap in excess of MWK 150 billion remains.

### Synthesis of results

Table 4 synthesizes the findings of the assessment in the benchmarking matrix. Introducing a universal NHI scheme in Malawi would increase revenues for health, but these would come predominantly from the formal sector and would be unlikely to cover the health sector funding gap. Targeting the informal sector for the purpose of revenue collection faces serious challenges, as MRA did not have the systems in place to collect revenue from informal sector businesses or individuals. The performance of existing poverty identification and targeting mechanisms was not commensurate with the requirements of a NHI scheme. Most likely, making NHI successful would require introducing a “service access fee” to incentivise enrolment, which cannot be recommended on equity grounds as it would likely lead to a serious deterioration in financial risk protection.

**Table 4:** Health financing benchmarking matrix

Health financing objective	Reform scenarios		
	MOH	NHI	Purchaser
Technical efficiency	No change expected, potential improvements depending on the outcomes of decentralization and hospital autonomy reforms	Potential improvements associated with more efficient purchasing e.g. improved treatment protocols, better financial management	Potential improvements associated with more efficient purchasing e.g. improved treatment protocols, better financial management
Equity	Improvement dependent on national rollout of national ID	User fees likely necessary to incentivize enrolment – reduction in access for poor because of dysfunctional targeting and identification systems in place. Potential improvements in service quality.	Potential improvements in service quality
Financial risk protection	No change expected	Depends on the introduction of user-fees and on the effectiveness of the identification of poor people and of the enrolment of informal sector populations; potential improvement through decreased reliance on informal payments	No direct impact; in the longer term, it can reduce providers’ reliance on user fees and informal payments
Policy coordination and resource allocation process	Depends on implementation of upcoming reforms e.g. revised EHP	Opportunities and challenges, outcome dependant on strengthening MOH stewardship position	Opportunities and challenges, outcome dependant on strengthening MOH stewardship position
Health outcomes	No change expected	Can improve outcomes through enforcement of standards of care and appropriate provider payment mechanisms	Can improve outcomes through enforcement of standards of care and appropriate provider payment mechanisms
Revenue mobilisation	Widening funding gap	Potential savings due to improved contracting + additional revenue from premium collection, partially offset by higher administration costs	No additional revenue collection mechanism, but potential savings due to improved contracting

## Discussion

### Summary of findings

The assessment identified the Purchaser Scenario as the most favourable reform model. It combines a higher net revenue effect than the NHI Scenario, while not creating the same negative equity and financial risk protection effects as the NHI model. Compared to the MOH scenario, it generates potential savings from efficiency gains while having a neutral to positive effect on equity.

At the same time, the analysis has made clear that the largest positive effects of reforms of the Purchaser Scenario (and the NHI Scenario) are gains from technical efficiency that rely on a number of accompanying reforms, most significantly accountable, improved and more autonomous management. However, implementing such reforms is challenging, both in technical and in change management terms; assumptions made when quantifying the effects of the reforms may not hold with partially or ineffectively implemented reforms. In this case, the additional complexity in policy coordination and resource allocation may be a serious drawback.

### Implications for policy

The chief policy recommendation emerging from the assessment was to focus initial reforms on the purchasing function as an essential first step to improving efficiency in the health sector. More broadly, moving towards strategic purchasing is not dependent on a health insurance design [11]. In Malawi, revenue collection could be added to the purchaser when it has developed its own capacities, and MRA has developed mechanisms to reach out to the informal sector. Establishing a full NHIS can remain a long-term objective of a health financing strategy. However, a premium-collecting NHIS would have no negative impact on equity only if targeting and administrative effectiveness were of very high quality. As long as this cannot be ensured, NHIS should not be attempted.

At the time of conducting this study, the Government of Malawi had started rolling out identity cards on a national scale and the process is ongoing [12]. A well-functioning national ID system linked with the health information system would support some of the challenges identified in this assessment, particularly in relation to targeting and ascertaining entitlement to health services. However, administering such ID systems incurs non-negligible costs – for example in Ghana, running the National Health Insurance Scheme ID system represents approximately 4% of the health insurance expenditure [13].

The assessment helped to structure the debate around NHI in Malawi by providing evidence based on a locally adapted model, taking into account a large amount of information, including on relevant issues such as local institutional dynamics, prevailing salary structures, capacities for enrolment and of poverty targeting schemes. With that, it allowed a much more nuanced understanding of the options available: following the recommendations of the assessment, policy makers started focusing on the potential benefits of strategic purchasing and have made this a priority for the future. A fiscal space analysis for Malawi’s health sector conducted approximately during the same time interval as our assessment found limited additional revenue to be gained from implementing a range of “innovative financing” options to raise additional revenue – essentially taxes on fuel and motor vehicle insurance – and also called for improving efficiency and sector governance [14].

At the same time, NHI remains a topic and is seen by many Malawian stakeholders as a necessary complement to other reforms. While private hospitals are able to provide good quality of care funded through medical insurance schemes and individuals willing and able to pay, public hospitals, particularly central hospitals, face immense pressure on services due to underfunding and overcrowding. Capturing a share of this market for the public health system through paying wings with improved (hotel) services in public hospitals is seen as a possible solution – and a formal sector NHI for paying-wing-based hospital services as a building block for the success of this policy reform.

### Lessons for other countries

The approach presented in this study could easily be applied to other countries and is flexible to be adapted to local needs and ideas about the design of different NHI options. Its breadth and depth allow a richer and more context-specific understanding of the quantitative and qualitative impacts of introducing NHI in a country than by previously developed tools such as SimIns [15]. It also provides an extension to predominantly qualitative [16] or case study-focused [17] feasibility assessments. The renewed interest of Malawian policy makers in an NHI for hospital-based-services (after having dropped the NHI-for-tertiary-care scenario from the assessment in Phase 2 of this assessment) shows that making health financing policy is an iterative process driven by several actors and various objectives.

Benchmarking tentative reforms against clear policy objectives using a combination of qualitative and quantitative approaches has the potential to improve coherence in decision-making but should be approached cautiously. Assessments of equally important objectives may well not be equally credible because of difficulties in identifying appropriate indicators and benchmark levels, measuring them with sufficient accuracy and obtaining relevant, context-specific, good quality data. For example, it would be difficult to specify a realistic, acceptable level of “technical efficiency” or “administrative complexity”. These challenges leave room for a degree of subjectivity in any such assessment (until they are overcome, including through further research), but they also create opportunities for meaningful, sustained stakeholder engagement throughout policy development and implementation.

### Limitations

The results of the quantitative modelling are only as good as the data available and the assumptions made. It is, therefore, crucial to embed the assessment into a process of stakeholder validation, especially if conducted by outside experts, as was the case in Malawi. Making quantitative comparisons across reform alternatives is particularly difficult. How would the status quo system develop in the absence of large-scale financing reforms, but with reforms in other health system building blocks being implemented? Similarly, the evidence base on the quantitative effects of health system efficiency improvement following the introduction of a purchaser-provider split remains limited, particularly in low-income settings with limited institutional capacities.

## Conclusion

As ever more countries look towards implementing national health insurance with a view to advancing towards universal health coverage, the proposed assessment framework can provide an orientation for evidence-informed health financing policies.

## Additional file

Additional file 1: **Appendix 1.** List of organisations consulted during the assessment phase (June – August 2016). **Appendix 2.** Further details on the health sector expenditure model. **Appendix 3.** Assumed staffing structures. **Appendix 4.** Details of NHIS revenue collection costs. (DOC 478 kb)

## Abbreviations

GDP: Gross Domestic Product
GIZ: Deutsche Gesellschaft für Internationale Zusammenarbeit
HICs: High-income countries
LMIC: Low- and middle-income countries
MWK: Malawi Kwacha
OPM: Oxford Policy Management
WHO: World Health Organization

## Footnotes

1 Programmes using the envisaged targeting mechanism are only present in 18 out of the 28 districts in Malawi and where they are in place, they only collect information from the 12.5 to 50% (depending on locality) of the population who are expected to be poor.

2 The three levies are: Malawi Rural Electrification Programme (MAREP), fuel storage levy and motor vehicle insurance levy.

## References

[1] World Health Organization. Global Health Expenditure Database. 2018. http://apps.who.int/nha/database/Select/Indicators/en. Accessed 29 Apr 2019.

[2] Schmidt J-O (2011). A scoping study on health insurance in Malawi.

[3] Ministry of Health (2014). Concept Paper on Health Insurance Scheme for Financing Malawi Health Sector.

[4] Ministry of Health (2014). Policy Reform Paper on Health Insurance for Financing Malawi Health Sector.

[5] DPP Party Manifesto. 2014. http://sdnp.org.mw/Elections_2014/manifesto/DPP-Manifesto-2014.pdf. Accessed 29 Apr 2019.

[6] Malawi Public Sector Reform Commission (2015). The president and Ministry of Health.

[7] Abiiro GA (2016). Estimating willingness-to-pay for health insurance in Malawi.

[8] Institute for Health Metrics and Evaluation (2016). GBD Compare Data Visualization.

[9] Giedion U, Alfonso EA, Diaz Y. The Impact of Universal Coverage Schemes in the Developing World: A Review of the Existing Evidence. 2013. http://siteresources.worldbank.org/HEALTHNUTRITIONANDPOPULATION/Images/IMPACTofUHCSchemesinDevelopingCountries-AReviewofExistingEvidence.pdf. Accessed 29 Apr 2019.

[10] Wang W, Temsah G, Mallick L (2016). The impact of health insurance on maternal health care utilization: evidence from Ghana, Indonesia and Rwanda. Health Policy Plan.

[11] World Health Organization (2017). Strategic purchasing for universal health coverage: unlocking the potential.

[12] United Nations Development Program. Over 9 million people register for National Identity Cards as Malawians make history. 2017. http://www.mw.undp.org/content/malawi/en/home/presscenter/articles/2017/12/05/over-9-million-people-register-for-national-identity-cards-as-malawians-make-history-.html. Accessed 29 Apr 2019.

[13] Wang H, Otoo N, Dsane-Selby L (2017). Ghana National Health Insurance Scheme: improving financial sustainability based on expenditure review.

[14] Chansa C, Mwase T, Matsebula TC, Kandoole P, Revill P, Makumba JB (2018). Fresh money for health? The (false?) promise of “innovative financing” for health in Malawi. Heal Syst Reform.

[15] WHO/GTZ. SimIns health financing policy tool. 2008. http://www.who.int/health_financing/tools/simins/en/. Accessed 29 Apr 2019.

[16] Zeng W, Kim C, Archer L, Sayedi O, Jabarkhil MY, Sears K (2017). Assessing the feasibility of introducing health insurance in Afghanistan: a qualitative stakeholder analysis. BMC Health Serv Res.

[17] Biggeri M, Nannini M, Putoto G (2018). Assessing the feasibility of community health insurance in Uganda: a mixed-methods exploratory analysis. Soc Sci Med.

