# Supplementary material for: Assessing the feasibility and appropriateness of introducing a national health insurance scheme in Malawi
Source: Glob Health Res Policy. 2019 May 20;4:13. doi: 10.1186/s41256-019-0103-5 (PMC6526615; doi:10.1186/s41256-019-0103-5)
Supplement: Supplementary file 1 — Appendix 1. List of organisations consulted during the assessment phase (June – August 2016). Appendix 2. Further details on the health sector expenditure model. Appendix 3. Assumed staffing structures. Appendix 4. Details of NHIS revenue collection costs. (DOC 478 kb) [file 41256_2019_103_MOESM1_ESM.doc]

**Appendix 1. List of organisations consulted during the assessment phase (June – August 2016)**

Airtel

Blantyre City Council

Christian Health Association of Malawi (CHAM)

Clinton Health Access Initiative (CHAI)

Daeyang Hospital

Department of the Registrar General

DFID

Farmers Union of Malawi

GIZ

Health and Rights Education Program (HREP)

Kamuzu Central Hospital

Malawi Confederation of Chambers of Commerce and Industry

Malawi Revenue Authority

Malawian Economic Justice Network (MEJN)

Medical Aid Society of Malawi (MASM)

Metropolitan Health (Division of MMI ZA)

Ministry of Health – Health Policy Unit

Ministry of Health – Planning and Policy Development

Ministry of Health – Central Monitoring and Evaluation Division

Ministry of Finance – Economic Planning and Development

Malawi Health Equity Network

Malawi Human Rights Commission

Ministry of Finance

Ministry of Foreign Affairs

Ministry of Trade and Industry

National Registration Bureau

National Statistics Office

Office of the Vice-President

Parliamentary Health Committee, Parliament of the Republic of Malawi

Queen Elizabeth Central Hospital – Blantyre

Reserve Bank of Malawi

Roads Fund Administration

Support for Service Delivery Integration (SSDI) Abt

Telecom Network of Malawi

Zomba Central Hospital

**Appendix 2. Further details on the health sector expenditure model**

The four NHI scenarios were implemented in the expenditure model as follows:

- Scenario 1 (MOH): assumes no changes in the way the health system operated during the last three years. This is a somewhat conservative assumption considering the reforms that are currently being considered (e.g. revisiting the essential health package, hospital autonomy). As such, there is potential to improve the effectiveness of resource allocation, however such effects could not be modelled given the early stage of planned reforms.
- Scenario 2 (NHI: purchasing agency and premium collection): a separate agency from the MOH would be created to identify the population covered, purchase services from providers, raise revenue and manage the overall health care provision with a view on long term efficiency for the outcomes to population health. Consequently, the running costs of a NHI agency are much higher than those of a purchasing agency (Scenario 4). The MOH governance and administrative costs are assumed to be lower than costs only for purchasing agency (i.e. diminishing savings, from 10% in year 1 to 3% in year 5[[1]](#footnote-2)). The costs of processing an increasing number of claims were assumed to increase expenditure on health governance and administration by 10% annually based on the consultant’s experience.

User fees were assumed to be introduced for the informal non-poor who are not enrolled in NHI i.e. MWK 1,000 for an outpatient visit and MWK 1,200 for an inpatient episode[[2]](#footnote-3). It was assumed that the introduction of user fees would reduce utilisation of outpatient visits by 15% in the first three years and 14% by year 5. Annual individual insurance premiums were assumed to be MWK 1,500 for outpatient care and MWK 1,500 for inpatient care[[3]](#footnote-4). It was assumed that the informal non-poor represent 20% of the population; by the end of year 5, 1% of the population would pay NHIS premiums (5% of the informal non-poor) and 19% would still pay user fees[[4]](#footnote-5).

The cost of collecting premiums from informal non-poor individuals was assumed to represent 25% of raised premiums, of which 10% are field collectors’ incentives and 15% are logistical costs (e.g. transport, security, administration). This is based on experience in other countries on health micro-insurance: 25% is higher than the recommended level of 20%[[5]](#footnote-6), but more aligned with the regional experience e.g. Tanzania CBHI has cost-to-revenue ratios in excess of 50%, albeit it has a voluntary component[[6]](#footnote-7). No communication campaign costs for NHI were factored in. Further, no administrative costs were assumed for collecting user fees by service providers.

- Scenario 3 (tertiary care, data not shown below as scenario was discarded in Phase 2): NHIS covers only inpatient care under a basic package for hospitalization for the population. This option would require similar administration as Scenario 2. However delinking tertiary care from secondary and primary care does increase the cost of tertiary care delivery[[7]](#footnote-8); the model assumed the cost of inpatient care increased by 10% in the first year (with cumulative increases in each subsequent year of 5%, 3%, 2% and 1%) and the number of outpatient visits would decrease (by 15% annually in the first two years and 14% annually in the following three years) informed by the consultants’ experience and evidence from the RSBY program in India[[8]](#footnote-9).
- Scenario 4 (purchasing agency): it is assumed the MOH restructures to purchase services from providers and enforces a minimal set of quality standards, leading to cost reductions due to purchasing activity and service improvements. Aggregate yearly cost reductions have been taken to be 3% for the first two years, dropping to 1% in year 5 – based on the consultant’s experience in Bangladesh. On the other hand, the costs of processing an increasing number of claims were assumed to increase the MOH expenditure on health governance and administration by 10% annually.

Table A2.1. Projected population growth

| **Population of Malawi** | | **Estimated population at Dec 31** | | | | **Projected population at Dec 31** | | | | |
| --- | --- | --- | --- | --- | --- | --- | --- | --- | --- | --- |
|  |  | **2012** | **2013** | **2014** | **2015** | **2016** | **2017** | **2018** | **2019** | **2020** |
| **Age** | *Total* | *16,035,385* | *16,559,037* | *17,101,849* | *17,630,162* | *18,030,681* | *18,431,195* | *18,831,715* | *19,232,229* | *19,632,747* |
| **0** |  | **643,019** | **620,964** | **596,855** | **567,691** | **556,337** | **545,210** | **534,306** | **523,620** | **513,148** |
| 1-19 |  | 8,486,455 | 8,817,532 | 9,157,920 | 9,542,121 | 9,656,848 | 9,771,347 | 9,885,624 | 9,999,681 | 10,113,526 |
| 20-59 |  | 6,148,235 | 6,354,584 | 6,571,109 | 6,800,531 | 7,075,496 | 7,350,460 | 7,625,425 | 7,900,387 | 8,175,353 |
| 60+ |  | 757,676 | 765,957 | 775,965 | 719,819 | 742,000 | 764,178 | 786,360 | 808,541 | 830,720 |
| Average age | | 21.05 | 20.99 | 20.97 | 20.67 | 20.96 | 21.23 | 21.48 | 21.73 | 21.97 |
| **Relative cost index** | | **0.88** | **0.87** | **0.86** | **0.85** | **0.85** | **0.85** | **0.85** | **0.85** | **0.85** |

Table A2.2. Projected health expenditure (MWK million)

|  | **2015/2016** | **2016/2017** | **2017/2018** | **2018/2019** | **2019/2020** | **2020/2021** | **2021/2022** |
| --- | --- | --- | --- | --- | --- | --- | --- |
| **Scenario 1 – MOH** |  |  |  |  |  |  |  |
| Total health expenditure | **380,471.4** | **454,273.4** | **517,512.4** | **574,656.0** | **634,615.1** | **698,103.7** | **766,141.6** |
| Per capita (MWK) | 21,580.7 | 25,194.5 | 28,078.1 | 30,515.3 | 32,997.5 | 35,558.1 | 38,243.5 |
| **Scenario 2 – Full NHI and user fees** |  |  |  |  |  |  |  |
| Total health expenditure, of which | **380,471.4** | **454,273.4** | **504,481.0** | **544,122.9** | **589,149.7** | **636,714.5** | **691,483.4** |
| Total NHI cost |  |  | 8,513.0 | 8,868.4 | 9,107.2 | 9,380.7 | 9,444.5 |
| NHI agency |  |  | 7,602.4 | 7,899.7 | 8,087.7 | 8,535.3 | 8,531.8 |
| Purchasing agency |  |  | 267.0 | 279.0 | 291.8 | 300.7 | 322.4 |
| Cost of NHI regulator |  |  | 12.9 | 13.3 | 13.9 | 15.0 | 16.1 |
| MRA NHIS department (formal sector) |  |  | 43.0 | 44.2 | 46.2 | 49.9 | 53.7 |
| NHIS individual premium collection (inside or outside MRA) |  |  | 138.2 | 153.6 | 169.7 | 186.7 | 205.0 |
| Additional health resources for processing (facility-level) |  |  | 449.4 | 478.7 | 497.9 | 293.1 | 315.4 |
| Per capita (MWK) | 21,580.7 | 25,194.5 | 27,371.0 | 28,894.0 | 30,633.5 | 32,431.3 | 34,516.8 |
| **Scenario 4 (purchasing agency)** |  |  |  |  |  |  |  |
| Total health expenditure, of which | **380,471.4** | **454,273.4** | **506,714.7** | **550,226.2** | **598,732.5** | **648,808.5** | **706,893.7** |
| Health governance and administration (other than MOH) |  |  | 449.4 | 478.7 | 497.9 | 293.1 | 315.4 |
| Purchasing agency |  |  | 267.0 | 279.0 | 291.8 | 300.7 | 322.4 |
| Per capita (MWK) | 21,580.7 | 25,194.5 | 27,492.2 | 29,218.1 | 31,131.7 | 33,047.3 | 35,286.0 |

Table A2.3. Projected revenues (MWK million)

|  | **2015/2016** | **2016/2017** | **2017/2018** | **2018/2019** | **2019/2020** | **2020/2021** | **2021/2022** |
| --- | --- | --- | --- | --- | --- | --- | --- |
| **Scenario 1 (MOH)** |  |  |  |  |  |  |  |
| **Total revenue, of which** | **331,340** | **364,277** | **392,135** | **416,997** | **442,802** | **469,848** | **498,559** |
| Government less earmarking of formal sector tax | 106,775 | 127,487 | 104,210 | 113,944 | 124,982 | 136,550 | 151,567 |
| Rest of World | 161,420 | 163,035 | 164,665 | 166,312 | 167,975 | 169,654 | 171,351 |
| Corporation | 11,333 | 13,237 | 14,759 | 16,048 | 17,361 | 18,716 | 20,137 |
| Households | 39,922 | 46,630 | 51,991 | 56,530 | 61,154 | 65,928 | 70,935 |
| NPISH | 11,890 | 13,888 | 15,485 | 16,837 | 18,214 | 19,636 | 21,127 |
| **Hypothetical options for raising revenue** |  |  |  |  |  |  |  |
| Public and private Employer/Employee contribution at 3%  each (100% NHIS membership)[[9]](#footnote-10) |  |  | 41,024 | 47,327 | 53,115 | 59,365 | 63,442 |
| Informal Sector Premium |  |  | 553 | 614 | 679 | 747 | 820 |
| Informal Sector User fees |  |  | 3,166 | 3,517 | 3,929 | 4,323 | 4,747 |
| Health fund (MAREP, storage levy and MV insurance – medium scenario) |  |  | 4,147 | 4,386 | 4,660 | 4,963 | 5,269 |
| **Scenario 2 (full NHI and user fees additional revenue)** |  |  |  |  |  |  |  |
| **Total revenue, of which** |  |  | **7,866** | **8,517** | **9,267** | **10,033** | **10,836** |
| User fees from informal non-poor |  |  | 3,166 | 3,517 | 3,929 | 4,323 | 4,747 |
| Premiums from informal non-poor |  |  | 553 | 614 | 679 | 747 | 820 |
| Health fund (MAREP, storage levy and MV insurance – medium scenario) |  |  | 4,147 | 4,386 | 4,660 | 4,963 | 5,269 |
| **Scenario 4 (purchasing agency)** |  |  |  |  |  |  |  |
| Savings from purchasing |  |  | 10,798 | 24,430 | 35,883 | 49,295 | 59,248 |

Table A2.4. Projected net revenues (MWK million)

|  | **2015/2016** | **2016/2017** | **2017/2018** | **2018/2019** | **2019/2020** | **2020/2021** | **2021/2022** |
| --- | --- | --- | --- | --- | --- | --- | --- |
| **Scenario 1 (MOH)** |  |  |  |  |  |  |  |
| Net revenue | -49,131 | -89,997 | -125,378 | -157,659 | -191,813 | -228,255 | -267,583 |
| Total expenditure | 380,471 | 454,273 | 517,512 | 574,656 | 634,615 | 698,104 | 766,142 |
| Total revenue | 331,340 | 364,277 | 392,135 | 416,997 | 442,802 | 469,848 | 498,559 |
| **Scenario 2 (full NHI and user fees)** |  |  |  |  |  |  |  |
| Net revenue | -49,131 | -89,997 | -117,030 | -135,608 | -164,523 | -192,748 | -228,620 |
| Total expenditure | 380,471 | 454,273 | 517,031 | 561,122 | 616,549 | 672,582 | 737,963 |
| Total revenue | 331,340 | 364,277 | 400,000 | 425,514 | 452,026 | 479,834 | 509,343 |
| **Scenario 4 (purchasing agency)** |  |  |  |  |  |  |  |
| Net revenue | -49,131 | -89,997 | -110,615 | -129,008 | -151,446 | -174,176 | -203,258 |
| Total expenditure | 380,471 | 454,273 | 506,897 | 550,391 | 598,907 | 648,987 | 707,086 |
| Total revenue | 331,340 | 364,277 | 396,282 | 421,383 | 447,462 | 474,811 | 503,828 |

Note: Total revenue for Scenario 2 was calculated as the sum of: total revenue for Scenario 1, revenue from public and private employee/employer contribution at 3% each and scenario

**Appendix 3. Indicative staffing structures**

Table A3.1. Indicative staffing structure of the NHI agency

| **Structure** | **Type of staff** | **2017/2018** | | **2018/2019** | | **2019/2020** | | **2020/2021** | | **2021/2022** | |
| --- | --- | --- | --- | --- | --- | --- | --- | --- | --- | --- | --- |
|  |  | **# staff** | **Salary** | **# staff** | **Salary** | **# staff** | **Salary** | **# staff** | **Salary** | **# staff** | **Salary** |
| **BOARD** |  | 1 | 37.5 | 1 | 41.0 | 1 | 44.0 | 1 | 47.0 | 1 | 51.0 |
| Ombudsman | Senior | 1 | 4.7 | 1 | 5.0 | 1 | 5.4 | 1 | 6.0 | 1 | 6.5 |
|  | Support | 2 | 3.5 | 6 | 4.0 | 7 | 4.3 | 8 | 40.0 | 10 | 5.4 |
| Internal Audit | Senior | 1 | 7.05 | 1 | 8.0 | 1 | 8.7 | 1 | 9.0 | 1 | 9.7 |
|  | Support | 3 | 3.5 | 15 | 4.0 | 22 | 4.3 | 28 | 5.0 | 34 | 5.4 |
| **EXECUTIVE** |  |  |  |  |  |  |  |  |  |  |  |
| Chief Executive | Executive | 1 | 9.4 | 1 | 10.0 | 1 | 10.8 | 1 | 12.0 | 1 | 12.9 |
|  | Support | 1 | 3.5 | 2 | 4.0 | 2 | 4.3 | 3 | 5.0 | 3 | 5.4 |
| Chief Financial Officer | Senior | 1 | 7.05 | 1 | 8.0 | 1 | 8.7 | 1 | 9.0 | 1 | 9.7 |
|  | Support | 3 | 3.5 | 24 | 4.0 | 28 | 4.3 | 35 | 5.0 | 42 | 5.4 |
| Actuary | Senior | 1 | 7.05 | 1 | 8.0 | 1 | 8.7 | 1 | 9.0 | 1 | 9.7 |
|  | Support | 1 | 3.5 | 2 | 4.0 | 2 | 4.3 | 3 | 5.0 | 3 | 5.4 |
| Chief Information Technology | Senior | 1 | 7.05 | 1 | 8.0 | 1 | 8.7 | 1 | 9.0 | 1 | 9.7 |
|  | Support | 5 | 4.7 | 10 | 5.0 | 13 | 5.4 | 16 | 6.0 | 20 | 6.5 |
| Research and policy | Senior | 1 | 4.7 | 1 | 5.0 | 1 | 5.4 | 1 | 6.0 | 1 | 6.5 |
|  | Support | 1 | 3.5 | 2 | 4.0 | 3 | 4.3 | 4 | 5.0 | 5 | 5.4 |
| Legal | Senior | 1 | 7.05 | 1 | 8.0 | 1 | 8.7 | 1 | 9.0 | 1 | 9.7 |
|  | Support | 1 | 3.5 | 2 | 4.0 | 3 | 4.3 | 4 | 5.0 | 5 | 5.4 |
| HR | Senior | 1 | 4.7 | 1 | 5.0 | 1 | 5.4 | 1 | 6.0 | 1 | 6.5 |
|  | Support | 3 | 3.5 | 5 | 4.0 | 6 | 4.3 | 7 | 5.0 | 8 | 5.4 |
| Facilities | Support | 5 | 3.5 | 18 | 4.0 | 20 | 4.3 | 25 | 5.0 | 28 | 5.4 |
| Transport | Driver | 5 | 2.8 | 19 | 3.0 | 22 | 3.2 | 28 | 3.0 | 33 | 3.2 |
| **OPERATIONS** |  |  |  |  |  |  |  |  |  |  |  |
| Chief Operating Officer | Senior | 1 | 7.05 | 1 | 8.0 | 1 | 8.7 | 1 | 9.0 | 1 | 9.7 |
|  | Support | 1 | 3.5 | 2 | 4.0 | 2 | 4.3 | 3 | 5.0 | 3 | 5.4 |
| Communication and education | Senior | 1 | 4.7 | 1 | 5.0 | 1 | 5.4 | 1 | 6.0 | 1 | 6.5 |
|  | Support | 3 | 3.5 | 8 | 4.0 | 10 | 4.3 | 12 | 5.0 | 14 | 5.4 |
| Client data management | Senior | 1 | 4.7 | 2 | 5.0 | 3 | 5.4 | 3 | 6.0 | 3 | 6.5 |
|  | Support | 20 | 3.5 | 210 | 4.0 | 230 | 4.3 | 260 | 5.0 | 295 | 5.4 |
| Processing payments to providers | Senior | 1 | 4.7 | 1 | 5.0 | 1 | 5.4 | 1 | 6.0 | 1 | 6.5 |
|  | Support | 9 | 3.5 | 45 | 4.0 | 47 | 4.3 | 52 | 5.0 | 56 | 5.4 |
| Medical reviews of service quality | Senior | 1 | 4.7 | 1 | 5.0 | 1 | 5.4 | 1 | 6.0 | 1 | 6.5 |
|  | Support | 3 | 3.5 | 20 | 4.0 | 23 | 4.3 | 27 | 5.0 | 33 | 5.4 |
|  | **TOTAL** | **81** |  | **406** |  | **457** |  | **532** |  | **609** |  |

Notes: This structure depicts a gradual, realistic deployment. In the health expenditure model it was assumed as 100% operational (2021/2022 levels) from year 1 (2017/2018). Salaries are annual (MWK million). Client data management assumes a target of 15 staff per district x 28 districts, plus 15 staff at HQ. Processing payments to providers assumes 2 staff per district x 28 districts. Medical reviews of service quality assumes 1 staff per district x 28 districts plus 5 staff at HQ. Transport assumes 1 driver per district x 28 districts plus 5 drivers at HQ. Audit, financial, HR and operations staff volumes informed by *Nemec J (2013) Outlining organizational set up for the National Health Insurance of Nepal along with its functions and functionaries*.

Table A3.2. Indicative staffing structure and costs of the purchasing agency (MWK million)

|  | **2017/2018** | | | **2018/2019** | | | **2019/2020** | | | **2020/2021** | | | **2021/2022** | | |
| --- | --- | --- | --- | --- | --- | --- | --- | --- | --- | --- | --- | --- | --- | --- | --- |
| **Staff** | **Number** | **Annual** | **Total** | **Number** | **Annual** | **Total** | **Number** | **Annual** | **Total** | **Number** | **Annual** | **Total** | **Number** | **Annual** | **Total** |
| Director | 1 | 4700 | 4,700 | 1 | 5110 | 5,110 | 1 | 5528 | 5,528 | 1 | 5959 | 5,959 | 1 | 6412 | 6,412 |
| Contracting | 38 | 3500 | 133,000 | 38 | 3806 | 144,628 | 38 | 4117 | 156,446 | 38 | 4438 | 168,644 | 38 | 4775 | 181,450 |
| Research | 2 | 3500 | 7,000 | 2 | 3806 | 7,612 | 2 | 4117 | 8,234 | 2 | 4438 | 8,876 | 2 | 4775 | 9,550 |
| Compliance | 6 | 3500 | 21,000 | 6 | 3806 | 22,836 | 6 | 4117 | 24,702 | 6 | 4438 | 26,628 | 6 | 4775 | 28,650 |
| Total cost of salaries | 47 |  | 165,700 | 47 |  | 180,186 | 47 |  | 194,910 | 47 |  | 210,107 | 47 |  | 226,062 |
| Rent of office |  |  | 16,570 |  |  | 18,019 |  |  | 19,491 |  |  | 21,011 |  |  | 22,606 |
| Other overhead cost |  |  | 24,855 |  |  | 27,028 |  |  | 29,237 |  |  | 31,516 |  |  | 33,909 |
| Vehicles | 6 |  | 16,570 | 6 |  | 18,019 | 6 |  | 19,491 | 6 |  | 21,011 | 6 |  | 22,606 |
| Training |  |  | 16,570 |  |  | 9,009 |  |  | 1,949 |  |  | 2,101 |  |  | 2,261 |
| Computers | 47 | $1,000 | 11,750 | 47 | $1,000 | 11,750 | 47 | $1,000 | 11,750 | 47 | $1,000 | 0 | 47 | $1,000 | 0 |
| Software and main frame computer | 1 | $100,000 | 15,000 |  |  | 15,000 |  |  | 15,000 |  |  | 15,000 |  |  | 15,000 |
| TOTAL ALL COST |  |  | 267,015 |  |  | 279,011 |  |  | 291,828 |  |  | 300,746 |  |  | 322,445 |

Note: staffing structure informed by *Nemec J (2013) Outlining organizational set up for the National Health Insurance of Nepal along with its functions and functionaries*.

**Appendix 4. Details of NHIS revenue collection costs**

The subsections below will look at only formal sector costs and benefits and then formal and informal combined.

It must be noted that these estimates presume that the MRA pre-planned and pre-budgeted investments in ICT and bottom-up data linkages on the informal sector (MSME business tracking etc.) are already paid for. As such this brings down the main costs for NHIS as it is of the opinion of MRA management that NHIS needs can be accommodated within the modernisation programme plans. It is also assumed that implementation is ongoing smoothly over the upcoming three-year period (2016-2018) and so by 2017/18 MRA are in a much stronger position to take on the NHIS premium collection. It is presumed that the second half of 2017 (of the 2017/18 fiscal year) is a planning and training stage.

- - 1. Formal Sector Only Cost Estimate

Administering NHIS premium collection for the formal sector only would be a simple and relatively cheap initiative for the MRA in setting up and implementation. From his experience the Human Resources Administration Manager in MRA has suggested the following set up as a minimum requirement:

- An NHIS department would be set up under the Domestic Taxes Division (see Figure C1):
  - NHIS department would require not more than ten new staff. MRA has around 1,300 staff nationally, some of which would be carrying out supporting tasks to the department (ICT, HR, Finance, etc.).
  - A Senior Manager, or Director, would head the department. The Director would report to the Commissioner of Domestic Taxes Division.
  - Nine Analysts are expected to be needed, and would report to the Director. These would be graduates with numeric backgrounds such as economics, finance, statistics, accountancy, or business degrees.
- No new technology is expected to be required, rather once the MRAs ITAS is fully operational the NHIS department would utilise data within these IT systems.
- Vehicles may be needed for the department to carry out checks as well as compliance and enforcement.

Emphasis would be placed on the analysis as this would be a new revenue collection area and all data would need to be carefully examined to ensure accurate collection and membership etc. Recruitment of graduates with solid numerical skills, experience in data analysis, and self-starters would be crucial to the success. This will be especially important given that there may be small iterative changes to certain aspects of the revenue collections in the immediate implementation phases.

Training would also be a core pre-implementation and ongoing expense. There would be a strong incentive to reduce human error on MRAs side (greatly assisted by the automation through ITAS) as well as ensure compliance through a solid communication strategy for NHIS members.

Figure C1: NHIS Formal-Sector-Only Department within the MRA Organisational Structure


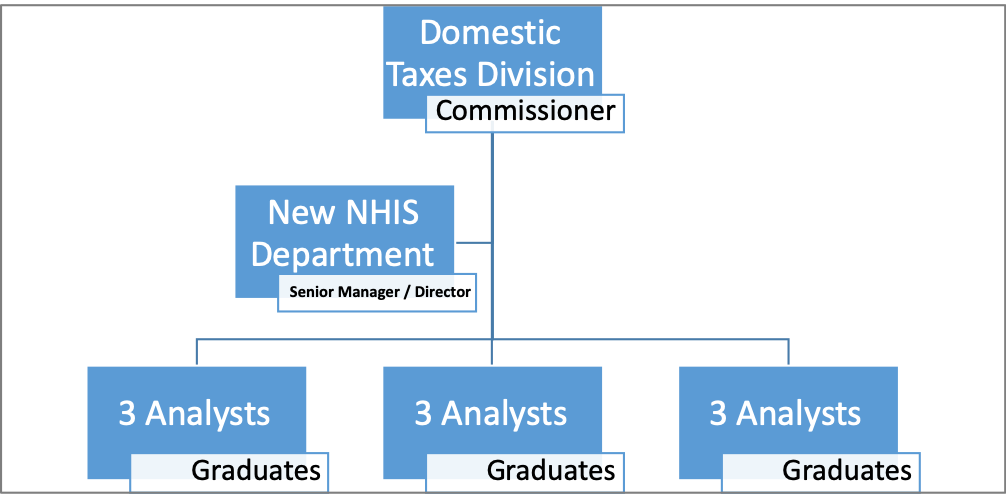


Source: Interview with MRA HR Department

How much this could cost has been estimated using the following methodology and underlying assumptions:

**Salaries** – Are based on data from the latest LFS carried out in 2013, inflated to 2017/18 through 2021/22 using estimates from the IMF WEO:

- *The Director’s Salary* is assumed to be around the level of the average monthly income of a tertiary educated Malawian. This was found to be 191,199 Kwacha in the 2013 LFS. Inflating this and moving to an annual basis gives an estimated salary of 4.7 million Kwacha in 2017/18 (just less than 6,000 USD a year which is almost 20 times the estimated GDP per capita in 2017/18).
- *An Analysts’ Salary* is assumed to be around the median monthly income of a tertiary educated Malawian. This was found to be 140,199 Kwacha in the 2013 LFS. Inflating this and moving to an annual basis gives an estimated salary of 3.5 million Kwacha in 2017/18 (just over 4,000 USD a year which is almost 15 times the estimated GDP per capita in 2017/18). The total cost for analysts will be nine multiples of this salary.

**Vehicle costs** – A public expenditure review focusing on travel costs found that travel costs are high in Malawi accounting for 12 to 14% of total expenditures on goods and services[[10]](#footnote-11). The majority of these costs are associated with internal Malawi travel - 80%. Fuel costs amount to 23% of domestic travel costs and are sensitive to fuel price changes. Vehicle maintenance is less than 20%. Vehicle purchases were found to be between 1.6 and 2% of recurrent expenditures within the total budget. Using these proportions as a basis the underlying assumption for the vehicle costs for NHIS department are as follows:

- *Year one* – Vehicle purchase cost of 2% of salaries (in lieu of recurrent expenditures, of which salaries make up the majority), plus, maintenance and fuel costs of 8% of salaries (in lieu of goods and services value).
- *Subsequent years* – Maintenance and fuel costs of 8% of salaries.

**Training** – The total training budget of the Ministry of Health has declined over recent years from 15% of operating costs in 2006/07 to 2% in 2011/12[[11]](#footnote-12). In 2011/12 the total training expenditure accounted for 8.3% of total goods and services national budget[[12]](#footnote-13). It is assumed the training needs would have to be front loaded in year one and will level off over the medium term:

- *Year one* – Training budget set at 10% of salaries. Will need specialist training on NHIS as well as MRA operations. This budget could also include a communication and sensitisation programme for the public.
- *Year two* – Training budget set at 5% of salaries. Expected to iron out implementation problems.
- *Subsequent years* – Training budget set at 1% of salaries as per TVET payroll levy requirement.

The total estimated cost over the next five years is shown in Table C1. Given the assumptions the NHIS department in charge of premium collection for the formal sector only would cost in the region of 43 million Kwacha in the first year, rising to 53.7 million over five years (mostly due to expected inflation). Investments in vehicle and training would be expected to decline over time. Analysts salaries would account for the largest proportion of spending – 78%.

Table C1: NHIS Formal-Sector-Only Department Cost Estimate (Million Kwacha)

|  | **2017/18** | **2018/19** | **2019/20** | **2020/21** | **2021/22** | **Share of Total Cost** |
| --- | --- | --- | --- | --- | --- | --- |
| Director | 4.7 | 5.1 | 5.6 | 6.0 | 6.5 | 12% |
| Nine Analysts | 31.1 | 34.0 | 36.8 | 39.8 | 42.8 | 78% |
| Vehicle - Purchase & Maintenance | 3.6 | 3.1 | 3.4 | 3.7 | 3.9 | 7% |
| Training | 3.6 | 2.0 | 0.4 | 0.5 | 0.5 | 3% |
| **TOTAL** | **43.0** | **44.2** | **46.2** | **49.9** | **53.7** | **100%** |

Source: Authors Calculations

Putting this into context, the total budget of MRA was 12.8 billion Kwacha in 2015/16 and 21.3 billion is planned for 2016/17[[13]](#footnote-14). Although the years do not align if we assume the MRA budget remained at 21.3 billion in 2017/18 plus the new NHIS budget, the NHIS department estimate accounts for 0.2% additional budget to the MRA. Other comparators[[14]](#footnote-15):

- As a proportion of the projected total government expenditure in 2017/18 this additional budgetary requirement accounts for 0.004%.
- As a proportion of the projected GDP this is 0.001%.

In sum, the additional cost requirement for administering the formal sector NHIS premium collections does not look to be substantial.

- - 1. Formal Sector and Informal Businesses Combined Cost Estimate

When we include the informal sector revenue collection requirements we see a different cost scenario. These costs will be significantly higher due to the challenges associated with identifying payees, tracking members, and overseeing compliance and enforcement. As such this would be labour-intensive and costly to collect. The following methodology and cost estimate is based on the assumption that the investments in ICT and bottom up data linkages to MSMEs and the informal sector individuals are operational.

A suggestion was made by MRA that the needs of the informal sector NHIS revenue collections may be along the lines of the existing project: Electronic Fiscal Devices (EFD). Box 1 provides some background information on this project. There are similar project features such as ensuring compliance in the informal sector, large staffing needs in the field, and so forth. Unfortunately, the full project information including costs was not shared with the consultant, as promised, by MRA. As such the NHIS formal and informal sector costing does rely on some information gained from interviews on this EFD project, but the core project costs were not available for assessment and possible replicated.

Box 1: Example of an MRA Informal Sector Project – Electronic Fiscal Devices

The Electronic Fiscal Device (EFD) project objective is to enhance revenue collections by ensuring tax compliance of VAT Registered taxpayers by enforcing the use of the Electronic Tax Registers.

The project makes it mandatory for every VAT taxpayer to procure, install and use EFD machines. Over the years, the main challenge in VAT administration has mainly been through tax evasion by non-issuance of tax invoices especially by small to medium taxpayers. It is hoped the introduction of EFDs will help curb this situation as sales transactions will now be monitored electronically, through a GPRS modem to the Authority’s central server. To further ensure compliance, the buyer will be encouraged to demand a tax invoice that will be generated by the EFDs as this is the only way a taxable transaction will be captured.

This project has been suggested as an appropriate project to base the NHIS costs on. The project looks at moving informal businesses into the formal sector, expanding the tax base by using electronic devices for VAT receipts. VAT project inspectors go into shops and see if shops using IT system and if people using receipts. This would be a similar scenario for NHIS premium collection requirements and enforcement. It has a department of 100 staff.

Source: MRA (2016) and Interview with MRA HR Department.

Inclusion of the informal sector would not change the placement of the NHIS department under the Domestic Taxes Division, as in the formal sector only scenario. However as can be seen in Figure C2 there would be a greater staffing need:

- The total number of staff would rise from ten to 100 (as per EDF staffing) under one director.
- The number of analysts would rise from nine to 15 at MRA headquarters; and
- There would be a need for field inspectors to carry out check across the country. The informal sector would mean many more people paying premiums under more complex and in more remote areas.
  - The 14 field inspection managers would each be responsible for two districts (28 districts in total). They would be responsible for the field inspection officers in their districts and report to the Director.
  - The 70 field inspector officers would be spread appropriately across the 28 districts. On average this provides 2.5 officers per district. They would report to their relevant field inspector managers.

Vehicle and training costs would rise in proportion to increased salary costs as per the methodology explained under the formal sector scenario.

Figure C2: NHIS Formal and Informal Department within the MRA Organisational Structure


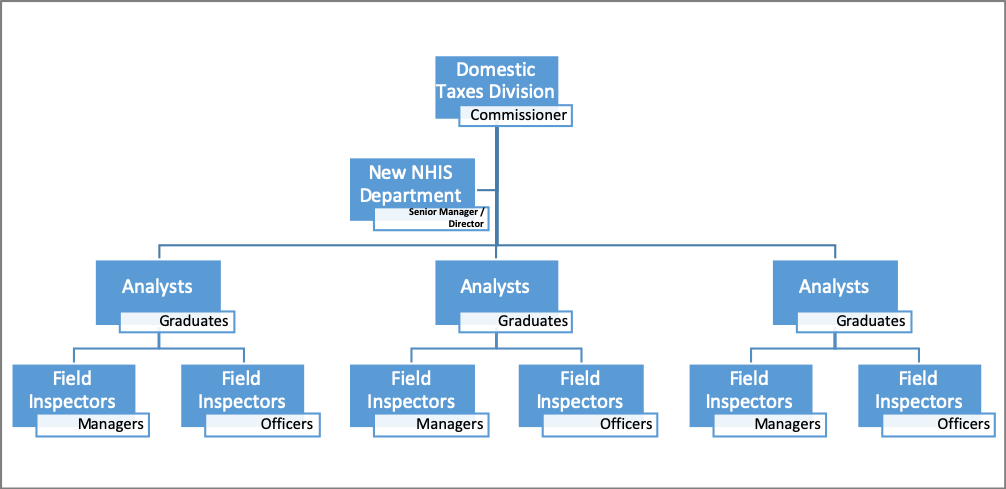


Source: Interview with MRA HR Department

The staffing numbers required to gather contributions from up-to-3 million informal sector workers are lower than expected due to the assumption that many will be able to use the new ICT systems such as the mobile phone app to pay their premiums[[15]](#footnote-16). There is much evidence across Sub Saharan Africa that mobile phone apps are widely used by the poor, these are briefly discussed in Box 2. With widening use of mobile phones and evidence of other countries successes a cost estimate with lower staffing needs seems appropriate. However, if the uptake of the new ICT / mobile phone app is not as high as expected the MRA enforcement costs will rise.

Box 2: Evidence on the Use of Mobile Phone Apps

In many Sub Saharan countries there are plans to develop various services accessed through mobile phones, such as mobile money. In other countries’ experience once available the demand for this type of service grows exponentially. There is much evidence across Sub Saharan Africa that mobile phone apps are widely used by the poor. For example:

Mobile banking is commonplace in Kenya, including transferring domestic remittances. This technological tool has been an important instrument in deepening financial services to the rural poor and the World Bank sees the use of ‘mobile money’ as a potential engine for growth and poverty reduction, estimating that, by end 2010, ‘15 million Kenyans (3/4 of the adult population) will use mobile money … transferring an estimated 7 billion USD annually (20% of GDP) by phone’.

Two further initiatives are mobile phone-based health and agricultural insurance. One such example is the *Kilimo Salama* crop insurance, which relies on weather station information and pays out direct to policy holders by mobile phones. Some of these initiatives are expected to lead to greater productivity in agriculture and have wider economic growth impacts.

Sources: Cited in MOHapatra and Ratha (2011), World Bank (2010), and http://opinionator.blogs.nytimes.com/2011/05/09/doing-more-than-praying-for-rain/

How much this could cost has been estimated using a similar methodology and underlying assumptions as the formal sector cost estimate. Set out below are the new or different assumptions relating to the informal sector:

**Salaries** – Again based LFS 2013 data, inflated to 2017/18 through 2021/22 using estimates from the IMF WEO:

- *Director and Analyst Salary* – Calculations remain unchanged other than the number of analysts rise from nine to 15.
- *Field Inspector Managers Salary –* is assumed to be around the average monthly income of a secondary educated Malawian. This was found to be 119,441 Kwacha in the 2013 LFS. Inflating this and moving to an annual basis gives an estimated salary of 3 million Kwacha in 2017/18 (just less than 4,000 USD a year which is 12 times the estimated GDP per capita in 2017/18). The total cost for field inspection managers will be 14 multiples of this salary.
- *Field Inspector Officers –* is assumed to be around the median monthly income of a secondary educated Malawian. This was found to be 28,667 Kwacha in the 2013 LFS. Inflating this and moving to an annual basis gives an estimated salary of 0.7 million Kwacha in 2017/18 (just less than 900 USD a year which is 3 times the estimated GDP per capita in 2017/18). The total cost for field inspection officers will be 70 multiples of this salary.

**Vehicles and Training** – Now the base for calculating these has risen from director and analyst salaries to include more analysts and all field inspectors. Thus raising the amount required. Vehicle and training costs will be significant as there will be a much greater need for outreach across the entire country. The costs may well rise to greater levels depending on how the MOH and MRA decide to allocate training requirements. MRA staff will need a strong comprehension of the NHIS and MRA operations but additionally the problems and challenges associated with willingness to pay and low compliance rates within the informal sector will need to be address both through training and greater enforcement.

The total estimated cost over the next five years is shown in Table C2Table C1. Given the assumptions the NHIS department in charge of premium collection for both the formal and informal sectors would cost in the region of 177 million Kwacha in the first year, rising to 221 million over five years (mostly due to expected inflation). Investments in vehicle and training would be expected to decline over time. Analysts’ salaries would account for the largest proportion of spending – 31% – closely followed by field inspection officers – 30%.

Table C2: NHIS Formal and Informal Department Cost Estimate (Million Kwacha)

| **Formal and Informal Sector** | **2017/18** | **2018/19** | **2019/20** | **2020/21** | **2021/22** | **Share of Total Cost** |
| --- | --- | --- | --- | --- | --- | --- |
| Director | 4.7 | 5.1 | 5.6 | 6.0 | 6.5 | 3% |
| 15 Analysts | 51.9 | 56.6 | 61.4 | 66.3 | 71.3 | 31% |
| Field Inspectors - 14 Managers | 41.3 | 45.0 | 48.8 | 52.7 | 56.7 | 25% |
| Field Inspectors - 70 Officers | 49.5 | 54.0 | 58.5 | 63.2 | 68.0 | 30% |
| Vehicle - Purchase & Maintenance | 14.7 | 12.9 | 13.9 | 15.1 | 16.2 | 7% |
| Training | 14.7 | 8.0 | 1.7 | 1.9 | 2.0 | 3% |
| **TOTAL** | **176.9** | **181.7** | **189.9** | **205.2** | **220.7** | **100%** |

Source: Authors Calculations

Putting this cost into context[[16]](#footnote-17):

- As a proportion of the MRA potential budget for 2017/18 the NHIS department estimate accounts for 0.8% additional budget to the MRA;
- As a proportion of the projected total government expenditure in 2017/18 this additional budgetary requirement accounts for 0.01%; and
- As a proportion of the projected GDP this is 0.004%.

The estimated cost of collecting premiums for the formal and informal sector is around four times more than the estimated cost of collecting for only the formal sector. This is expected to be a minimum scenario. As a proportion of the MRAs existing budget, government total expenditures and GDP these values are not debilitating. However, the value for money and / or effective use of funds will need to be ensured though the success of collecting the revenues. This depends on the pre-planned and budgeted investments made by MRA (ICT and bottom up data on the informal sector). If these are implemented and can be relied upon to accurately identify and track informal sector workers as NHIS members, then the collections could be accomplished with relative ease. Relative, given the large resources required in terms of staffing etc. to collect from informal sector. It is seen as a manageable task by MRA but one that will require huge investment, rethinking and re-strategizing.

- 1. Potential Revenue Collection

Estimate of the total potential revenues from the formal and informal sector are set out below along with the methodology.

Table C3 provides the maximum potential number of members the NHIS could gain; i.e. the total number of employed people in Malawi. This is based on the LFS 2013 information for formal public, formal private and informal employees. A 50:50 split was assumed for the poor and non-poor within the informal sector[[17]](#footnote-18). This is based on three sources, namely the LFS 2013, IHS 2011 and the FinScope Survey. Each have various data point to the fact that half of the informal sector are on or below the poverty line.

It must also be noted that this revenue collection estimation is based on the total numbers of employees in the informal sector, not just the registered businesses. At present, due to problems in data collection as mentioned above, there is a lack of information about the number of informal businesses in Malawi. But with the planned improvements MRA should be able to track businesses in the future, but not individuals. The World Bank estimate that most of the informal sector businesses are sole traders, and working in the agriculture sector[[18]](#footnote-19). Therefore, of the total number of informal non-poor it is likely that they will be sole traders in the agriculture sector, and potentially not markedly different in number as the number of informal non-poor. With this in mind we use the total number of informal non-poor as a maximum representative for those the MRA could track through business registration.

Table C3: Potential Membership (Millions of Employees)

|  | **2017/18** | **2018/19** | **2019/20** | **2020/21** | **2021/22** |
| --- | --- | --- | --- | --- | --- |
| **Total Employed** | **6.6** | **6.9** | **7.4** | **7.8** | **8.3** |
| ***Formal*** | ***0.7*** | ***0.8*** | ***0.8*** | ***0.9*** | ***0.9*** |
| Public | 0.2 | 0.2 | 0.2 | 0.3 | 0.3 |
| Private | 0.5 | 0.6 | 0.6 | 0.6 | 0.7 |
| ***Informal*** | ***5.8*** | ***6.2*** | ***6.5*** | ***6.9*** | ***7.3*** |
| Non-Poor | 2.9 | 3.1 | 3.3 | 3.5 | 3.7 |
| Poor | 2.9 | 3.1 | 3.3 | 3.5 | 3.7 |

Source: Projections based on formal and informal split from LFS 2013, poor and non-poor are author’s calculations based on evidence in HIS (2010/11) and FinScope (2012).

Average salaries for each type of employee are required to calculate the potential premium. These were assumed as follows:

- ***Formal Public*** – i.e. Civil Servants. Value of the wages and salaries budget line was taken from the 2017/18 and 2018/19 budgets and divided by the number of people employed in the formal public sector as in Table C3. Projections for 2019/20 to 2021/22 used an annual growth rate based on inflation rate from IMF WEO. Gives an average annual salary of 1.5 million Kwacha in 2017/18 (1,817 USD).
- ***Formal Private*** – The average salary was assumed as equal to the mean wage of a secondary educated Malawian, which was found to be 0.3 million Kwacha in the 2013 LFS. Inflated to 2017/18 prices this is now 0.7 million Kwacha a year (888 USD).
- ***Informal Non-Poor*** – The average salary was assumed as equal to the mean wage of those living in an urban area, which was found to be 0.2 million Kwacha in the 2013 LFS (relevant as in chapter 2 urban residents are more likely to be better paid if in the informal sector). Inflated to 2017/18 prices this is now 0.5 million Kwacha a year (638 USD).
- ***Informal Poor*** - The average salary was assumed as equal to the median wage of those with no education which is also in-line with the median wage of rural residents (relevant as in chapter 2 rural people with no education are more likely to be in the informal sector around the poverty line). These are both found to be 0.1 million Kwacha in the 2013 LFS. Inflated to 2017/18 prices this is now 0.3 million Kwacha a year (372 USD).

Each salary was inflated over the five-year time line using the IMF WEO projections.

Contributions can then be calculated by multiplying the number of employed / potential members, with the average salaries, and by the premium rate of 3% for employees and employers. Table C4 provides the findings from this methodology, to clarify:

- ***Formal Public*** – Civil Servants pay 3% of their salary and the government (employer) pays 3%.
- ***Formal Private*** – Employee and employer both pay 3% of the employee’s salary.
- ***Informal Non-Poor*** – Pay 3% of their estimated average salary.
- ***Informal Poor*** – Do not pay as they are unable, by definition they are around or under the poverty line. In this scenario the government pays a subsidy but this is above the informal poor average salary. Instead it is equal to the informal non-poor average salary – 3% is paid. This is to ensure adequate funds are available in the NHIS for the poor to received adequate health care.

Table C4: Potential Contributions (Million Kwacha)

|  | **2017/18** | **2018/19** | **2019/20** | **2020/21** | **2021/22** |
| --- | --- | --- | --- | --- | --- |
| **Total** | **129,654** | **149,835** | **170,844** | **194,160** | **206,324** |
| **Formal** | **41,024** | **47,327** | **53,115** | **59,365** | **63,442** |
| Public | 18,780 | 21,600 | 23,568 | 25,535 | 27,582 |
| Private | 22,244 | 25,727 | 29,547 | 33,830 | 35,860 |
| **Informal** | **88,630** | **102,509** | **117,729** | **134,795** | **142,882** |
| Non-Poor | 44,315 | 51,254 | 58,864 | 67,397 | 71,441 |
| Poor | 44,315 | 51,254 | 58,864 | 67,397 | 71,441 |

Source: Authors calculations from various data sources as described above

The scenario set out in Table C4 is an optimistic scenario where all employees in Malawi are paying NHIS premiums. This would require a mandatory membership decision from the MOH / Government.

- 1. Benefits versus Costs

Table C5 considers the estimated costs and potential benefits as calculated above. It is clear that the potential premiums to be collected for NHIS seriously outweigh the estimated costs. Indeed, the costs are far less than 1% of the projected revenues. However, there are two important elements to note:

- The costs are a minimal estimate as they do not include MRA overheads;
- The premiums are a maximum estimate as they assume membership is 100% of the eligible employed population.

Table C5: Comparing Estimated Costs and Potential Benefits (Million Kwacha)

|  | **2017/18** | **2018/19** | **2019/20** | **2020/21** | **2021/22** |
| --- | --- | --- | --- | --- | --- |
| Estimated Formal and Informal Costs | 177 | 182 | 190 | 205 | 221 |
| Estimated Premium Collections | 129,654 | 80,614 | 89,181 | 98,322 | 102,398 |
| **Costs as % Benefits** | **0.1%** | **0.2%** | **0.2%** | **0.2%** | **0.2%** |

Source: Authors calculations

Therefore, a sensitivity analysis was carried out as shown in Table C6. Some key assumptions are changed as follows:

- The costs are raised by 50%. This could be an inclusion of MRA overhead cost of around 20% of the department costs, and raising of training and enforcement costs in the event that more is needed on an ongoing annual basis.
- On the premium collection side, the membership is reduced across the board to 50% of the potential members (i.e. 50% of employed people), and the government pays no subsidy for the informal poor.

Within these assumptions the costs are still significantly less than the potential value of the premiums collected.

Table C6: Sensitivity Analysis for Costs and Benefits (Million Kwacha)

|  | **2017/18** | **2018/19** | **2019/20** | **2020/21** | **2021/22** |
| --- | --- | --- | --- | --- | --- |
| **Costs - 50% Overheads, Training & Enforcement** | **265** | **273** | **285** | **308** | **331** |
| **Benefits - 50% membership & Zero Subsidy** | **52,059** | **60,091** | **67,774** | **76,149** | **81,232** |
| ***Formal*** | ***29,902*** | ***34,463*** | ***38,342*** | ***42,450*** | ***45,512*** |
| Public | 18,780 | 21,600 | 23,568 | 25,535 | 27,582 |
| Private | 11,122 | 12,863 | 14,773 | 16,915 | 17,930 |
| ***Informal*** | ***22,158*** | ***25,627*** | ***29,432*** | ***33,699*** | ***35,721*** |
| Non-Poor | 22,158 | 25,627 | 29,432 | 33,699 | 35,721 |
| Poor | 0 | 0 | 0 | 0 | 0 |
| **Costs as % Benefits** | **0.5%** | **0.5%** | **0.4%** | **0.4%** | **0.4%** |

Source: Authors calculations

To put into context, the baseline scenario net contributions (premiums collected minus costs) would equate to the following[[19]](#footnote-20):

- The 2017/18 net contribution would equate to 80% of the projected government’s spending on health; and
- The 2017/18 net contribution would be enough to cover only 22% of the total health expenditure needs.

Therefore, although the net outcome is positive, the nominal value of potential contributions remains low relative to healthcare costs in Malawi. Therefore, much more work would need to be carried out on appropriate premium rates (potentially disaggregating by salaries) and their affordability.

As said previously, these estimates and projection rely on the assumption that MRA investments are pre-planned and budgeted, and will be implemented within the three-year timeline to satisfaction. No ICT implementation costs are appropriated to the NHIS costs and no costs are included for the work being carried out to gain better access to informal sector data (bottom up linkages). These assumptions allow such a positive projection to be made.

1. Based on the consultant’s experience. [↑](#footnote-ref-2)
2. Expert consultant’s assessment of plausible levels for Malawi. [↑](#footnote-ref-3)
3. Based on other countries’ experience of 1% of average per capita income. [↑](#footnote-ref-4)
4. Coverage levels assumed based on the consultants’ experience of micro health insurance roll-out. [↑](#footnote-ref-5)
5. Wipf J and Garand D (2010) Performance indicators for microinsurance. A Handbook for Microinsurance Practitioners. 2^nd^ edition. [↑](#footnote-ref-6)
6. Borghi J, Makawia S, Kuwawenaruwa A (2015) The administrative costs of community-based health insurance: a case study of the community health fund in Tanzania. Health Policy and Planning, 30(1): 19-27. [↑](#footnote-ref-7)
7. Nitin Shrivastava, Jeanna Holtz and Alice Merry (2014) Providing outpatient insurance to complement Rashtriya Swastya Bima Yojana (RSBY) Final Report OPD Project Puri and Mehsana. ICICI Lombard. http://www.impactinsurance.org/projects/lessons/providing-outpatient-insurance-complement-rsby

   http://www.impactinsurance.org/sites/default/files/Final%20report%20OPD.pdf [↑](#footnote-ref-8)
8. Shoree S, Sharma S and Ruchismita R (2014) Outpatient care in RSBY: A study of program’s pilot experiments. Research Paper #43. Impact Insurance, available at <http://www.impactinsurance.org/publications/rp43> [↑](#footnote-ref-9)
9. In effect, earmarking of government tax. Assumption made in the original SSDI model. It is not calculated as such to actuarially balance the cost of the benefit package as the package has not yet been defined. [↑](#footnote-ref-10)
10. Travel PER (2010) cited in World Bank (2013). [↑](#footnote-ref-11)
11. Ibid. [↑](#footnote-ref-12)
12. Ibid. [↑](#footnote-ref-13)
13. Ministry of Finance (2016b). [↑](#footnote-ref-14)
14. Total Government Expenditure and GDP projections from IMF WEO. [↑](#footnote-ref-15)
15. Labour Force Survey 2013 found that 5.8 million were employed in the informal sector, and Integrated Household Survey (2010/11) and FinScope Survey (2012) both found 50% of informal sector workers were poor. Therefore, at most there will be 3 million non-poor informal sector workers. [↑](#footnote-ref-16)
16. MRA budget based on estimate given in MOF Budget for 2016/17, Total Government Expenditure and GDP projections from IMF WEO. [↑](#footnote-ref-17)
17. Data from the Labour Force Survey (2014), Integrated Household Survey (2010/11) and the FinScope survey (2012) suggest that half of the informal sector are poor. [↑](#footnote-ref-18)
18. World Bank Survey on Micro Small and Medium Businesses (2012). [↑](#footnote-ref-19)
19. Projections for government spending on health and total health needs are sourced from the OPM Actuarial Model for the wider report on NHIS. [↑](#footnote-ref-20)
